# Exploring the possible mechanism of low-dose naloxone exposure improving the immune microenvironment of gastric cancer tumors

**DOI:** 10.3389/fimmu.2025.1524930

**Published:** 2025-03-26

**Authors:** Xiangzhen Min, Yan Ma, Mingyue Lv, Xiaoxi Li, Renjun Lv, Xiaoyong Zhao, Yufang Leng

**Affiliations:** ^1^ The First School of Clinical Medicine, Lanzhou University, Lanzhou, China; ^2^ Department of Anesthesiology, Shandong Cancer Hospital and Institute Affiliated to Shandong First Medical University (Shandong Academy of Medical Science), Jinan, Shandong, China; ^3^ Department of Anesthesiology, Affiliated Hospital of Shandong University of Traditional Chinese Medicine, Jinan, Shandong, China; ^4^ Department of Pharmacy, Affiliated Hospital of Shandong University of Traditional Chinese Medicine, Jinan, Shandong, China; ^5^ Department of Anesthesiology, The First Hospital of Lanzhou University, Lanzhou, Gansu, China

**Keywords:** low-dose naloxone, immune function, CD8+T cells, immune checkpoint, opioid receptors

## Abstract

**Introduction:**

Gastric cancer, one of the most common cancers of the digestive tract, has high incidence and mortality rates. Until recently, surgery has been the most effective method of treatment for gastric cancer. Surgery, however, inevitably results in dysfunction of the autonomic nervous system, entry of tumor cells into the bloodstream, and immunosuppression during the perioperative period, all of which increase the risk of complications in patients with gastric cancer. Opioid receptors play an important role in the proliferation and secretion of cytotoxic factors by immune cells. Opiate usage inhibits immune cell function, reduces the release of cytotoxic factors, and enables tumor cells to evade the immune system, thereby increasing the risk of perioperative complications. Opioid antagonists may reverse opioid-mediated immunosuppression in several ways. However, studies on the molecular biology of opioid receptor antagonists in relation to their ability to improve immune function in patients with gastric cancer are limited.

**Methods:**

We first analyzed the cancer genome atlas stomach adenocarcinoma (TCGA-STAD) dataset to determine the correlation between changes in immune function and toll-like receptor 4 (TLR4) expression in patients with gastric cancer. A transwell co-culture system was established using CD8^+^T and mouse forestomach carcinoma (MFC) cells. CD8^+^T cells were treated with different concentrations of naloxone to determine the most effective concentration for killing the tumor cells. We then performed western blotting and quantitative realtime polymerase chain reaction to determine the expression of lymphocyte activation gene 3 (Lag3), perforin 1 (Prf1), programmed death ligand 1 (PD-1), T-cell immunoglobulin and mucin domain 3 (TIM-3), and TLR4/AKT/mTOR in CD8^+^ T cells. An MFC-derived allograft mouse model was used to study the *in vivo* changes in the immune cells. Flow cytometry, ELISA, WB, and PCR were used to examine changes in the number of immune cell populations in the spleen, secretion of cytotoxic factors by immune cells, opioid receptors, AKT/mTOR, and immune checkpoint proteins, respectively, in CD8^+^T cells.

**Results:**

We found that changes in perioperative immune function strongly correlated with TLR4 expression on the surface of immune cells in patients with gastric cancer. Low-dose naloxone (LDN) increased CD8^+^ T cell cytotoxicity, inhibited CD8^+^ T cell exhaustion, inhibited Lag3, Prf1, and Tim3 expression, and increased AKT and mTOR expression in CD8^+^ T cells. Opioid receptors were downregulated in CD8^+^ T cells following LDN administration.

**Conclusion:**

LDN improved the ability of CD8^+^T cells to kill gastric cancer cells and reduced CD8^+^T cell exhaustion. The mechanism underlying these LDN-mediated phenomena may involve regulation of immune checkpoint expression in CD8^+^ T cells, increased cytotoxic factor secretion by CD8^+^ T cells via the TLR4/AKT/mTOR pathway, or regulation of expression of opioid receptors on CD8^+^T cells, thereby further affecting CD8^+^T cell exhaustion.

## 1 Introduction

According to the World Health Organization, gastric cancer ranks fourth in terms of the incidence rate and third in terms of mortality (1). Thus, gastric cancer presents a significant threat to human life and health globally. Approximately 239,000 people die annually from stomach cancer in China (2). Comprehensive surgical treatment has been the main treatment for patients with stages 0, I, II, and III gastric cancer. However, surgery-associated factors, including surgical trauma, blood loss, and anesthesia, severely affect the patient’s immune system (3). Furthermore, despite its effectiveness, surgery is associated with high pain levels, severe stress responses, increased inflammation, decreased immunity, and high rates of pulmonary complications. Postoperative recovery is often hindered by these conditions, and several factors contribute to the underlying mechanisms. Optimizing anesthetic choice and quality has been found to play an increasingly important role in recovery (4). Currently, immune dysfunction is the most pressing problem faced by clinicians in the postoperative care of patients with gastric cancer.

The human opioid receptor family consists of four G protein-coupled receptors (μ, δ, κ, and ζ), with μ receptors constituting the main opioid receptors (5, 6). The ζ-receptor is also known as the opioid growth factor receptor (OGFr), and is expressed on or in immune cells, which suggests that agonists and antagonists of the ζ-receptor may have an immunoregulatory effect. The opioid receptors are activated following binding of different types of opioid ligands and substances, which activate the G protein-coupled receptors on the inner side of the plasma membrane. This leads to the activation of numerous downstream signaling factors, regulation of membrane ion channels, and nuclear transcription. Opioid drug-mediated activation of extracellular signal-related kinases results in increased release of arachidonic acid, and the expression of the oncogenes c-FOS and jun-B is upregulated, which is associated with immune regulation via reduced DNA binding ability of the transcription factors activator protein-1 and nuclear factor κB (7). Additionally, increasing evidence (8–10) suggests that opioids may negatively impact the immune system, progression of cancer, metastasis, and recurrence of cancer. However, little is known about the molecular and biological mechanisms by which opioid receptors affect perioperative immunity in patients with cancer.

Naltrexone, a non-selective opioid receptor antagonist, is primarily used in rehabilitation therapy of opiate addicts following discharge from a rehabilitation program to eradicate their addiction, maintain a normal lifestyle, and prevent relapse (11). Several major findings (12, 13) on off-label naltrexone use have been reported in recent years. Within a specific dosage window, naltrexone can be used to treat many autoimmune and malignant diseases, and alleviate mental illness symptoms. Increasing evidence indicates (14, 15) that naltrexone binds to opioid receptors in immune and tumor cells to exert its immunoregulatory effects. Based on these new discoveries, naltrexone represents a promising immunomodulatory agent for the treatment of cancer and other immune-related diseases. In a previous study (16), peripheral opioid receptor antagonists were shown to help patients recover from T-cell exhaustion, promote tumor-killing system activity, and improve prognosis. Further studies (17, 18) have shown that low-dose naloxone (LDN) appears to work as an immunomodulator by directly binding to OGFr in immune cells. LDN simultaneously blocks toll-like receptor 4 (TLR4) in macrophages and microglia. LDN also exerts its anti-inflammatory effects through non-opioid antagonist pathways (19).

However, whether LDN can improve the immune function in postoperative patients with gastric cancer remains unclear. Therefore, the purpose of the present study was to investigate the specific molecular mechanisms by which LDN affects immune cells using *in vitro* and mouse forestomach carcinoma (MFC) tumor-bearing mouse models.

## 2 Materials and methods

### 2.1 Analysis of the correlation between changes in immune cell function and TLR4 expression in gastric cancer

The cancer genome atlas stomach adenocarcinoma (TCGA-STAD) dataset was sourced directly from the Genomic Data Commons (GDC) portal. Our study included the transcriptome transcript per million (TPM) expression data from 373 cancer samples. To estimate the immune cell fractions, we applied the R package “Immunedeconv” to the integrated TPM data. After obtaining immune cell infiltration results for each patient, we conducted a Pearson correlation test to analyze the relationship between the proportion of immune cells and TLR4 expression levels. Statistical significance was set at p < 0.05.

### 2.2 Drugs and key chemicals

Naloxone hydrochloride injection was purchased from Beijing Huasu Pharmaceuticals Co. Ltd(X-100). Cell Counting Kit-8 (CCK-8) was purchased from Wuhan Saiwei Biotechnology Co. Ltd(G4103). CD8^+^T cells and CD8^+^T cell complete culture medium were purchased from Bluefcell(BFN60810741). Annexin V-PE/7-AAD Apoptosis Assay Kit was purchased from Meilunebio(MA0429). Enzyme-linked immunosorbent assay (ELISA) kits for human lactate dehydrogenase (LDH)(F0350-B), tumor necrosis factor-α (TNF-α)(F0121-B), interleukin-6 (IL-6)(F0049-B), IFN-γ(F0033-B), and Granzyme B(F0121-B) were purchased from Shanghai Fanke Biological Technology Co. Ltd. Antibodies against T cell immunoglobulins and mucin domain-containing protein 3 (TIM-3) were purchased from Abcam(ab185703). Super Pure RNA Extraction Kit and TRIzol^®^ Reagent were purchased from Jiangsu Cowin Biotech Co. Ltd(CW0581M). SYBR^®^ Green Premix Pro Taq HS qPCR Kit (AG11701) and Evo M-MLV RT Premix (AG11706)for qPCR were purchased from Accurate Biotechnology (Hunan) Co. Ltd. Antibodies against TLR4(66350-1-Ig), p-AKT(66444-1-Ig), and p-mTOR(67778-1-Ig) were purchased from Proteintech. PE anti-mouse CD4 (116005), FITC anti-mouse CD8a(100803), PE anti-mouse F4/80(123109), FITC anti-mouse CD86(105005), and FITC anti-mouse CD206 (MMR)(141703) antibodies were purchased from Biolegend. PCR primers were designed using a primer design tool (http://www.ncbi.nlm.nih.gov/tools/primer-blast/), and are listed in **Supplementary Table S1**.

### 2.3 Experimental design

#### 2.3.1 *In vitro* studies

**Figure 1:**
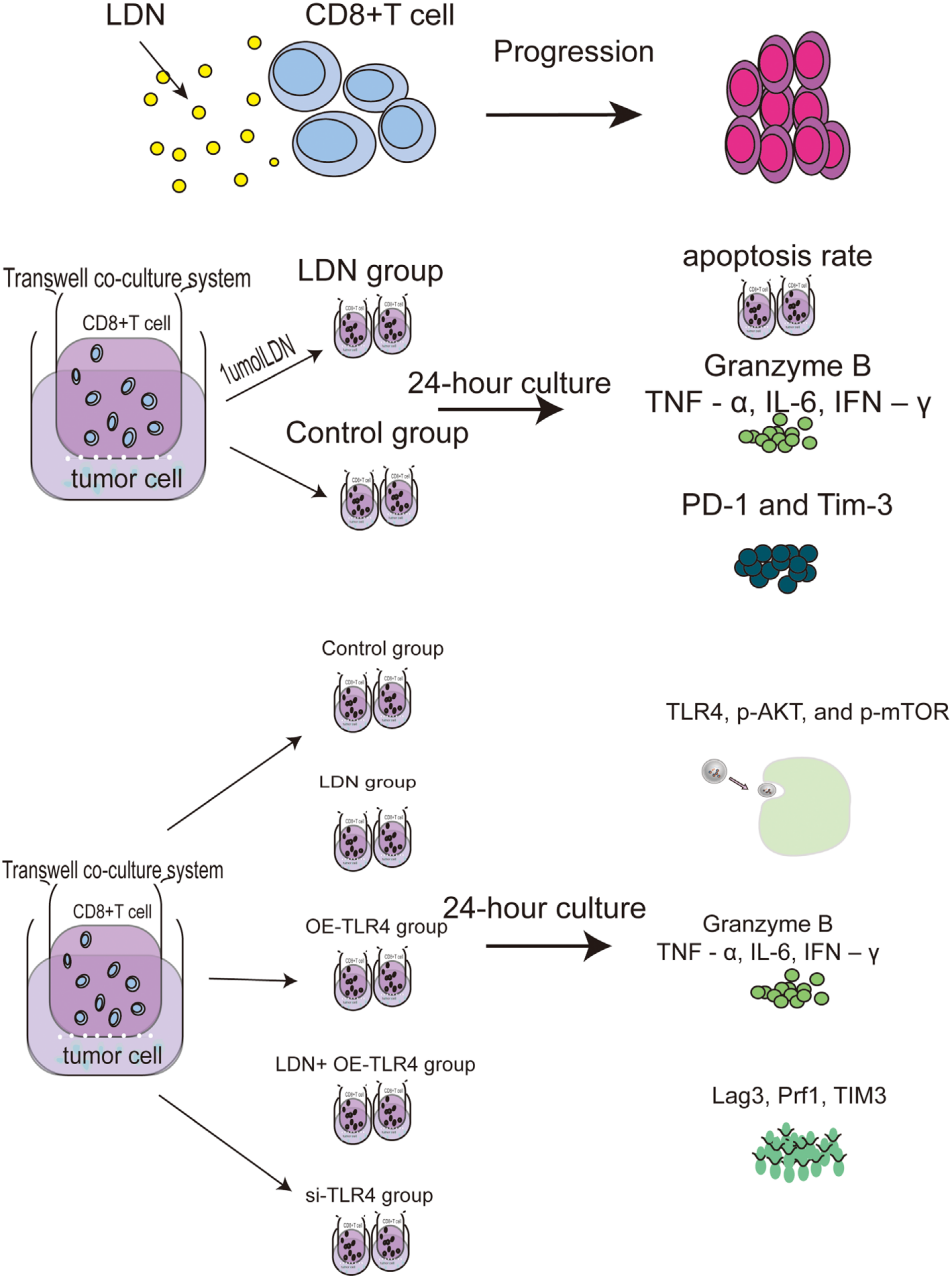
Flowchart of the cell experiments.

(a) CD8+T cells were treated with different concentrations of naloxone (10 nmol, 100 nmol, 1 µmol, 10 µmol, and 100 µmol). A CCK-8 kit was used to analyze the effect of naloxone on CD8+T cell proliferation. (b) CD8+T and MFC cells were used to establish a transwell co-culture system. The cultures were randomly divided into two groups: control and LDN group. The control group received no treatment, whereas the LDN group was treated with 1.0 µmol LDN. The LDH content in the supernatants from the two MFC groups was analyzed after 24 h of cultivation. The apoptotic rate and expression levels of PD-1 and TIM-3 in CD8+T cells was measured. The levels of TNF-α, IL-6, IFN-γ, and Granzyme B (GZMB) in the supernatant of CD8+T cells was measured. (c) CD8+T and MFC cells growing in transwell co-culture systems were randomly divided into control (no special treatment), LDN (CD8+T cells treated with 1.0 µmol LDN), OE-TLR4 (TLR4 overexpression in CD8+T cells), LDN + OE-TLR4 (CD8+T cells overexpressing TLR4 and treated with 1.0 µmol LDN), and si-TLR4 (siRNA mediated TLR4 knockdown in CD8+T cells) groups and treated accordingly. Next, the expression levels of Lag3, Prf1, TIM3, TNF-α, IL-6, IFN-γ, TLR4, p-AKT, and p-mTOR were measured in CD8+ T cells in each group (**Figure 1**).

#### 2.3.2 Animal studies

**Figure 2:**
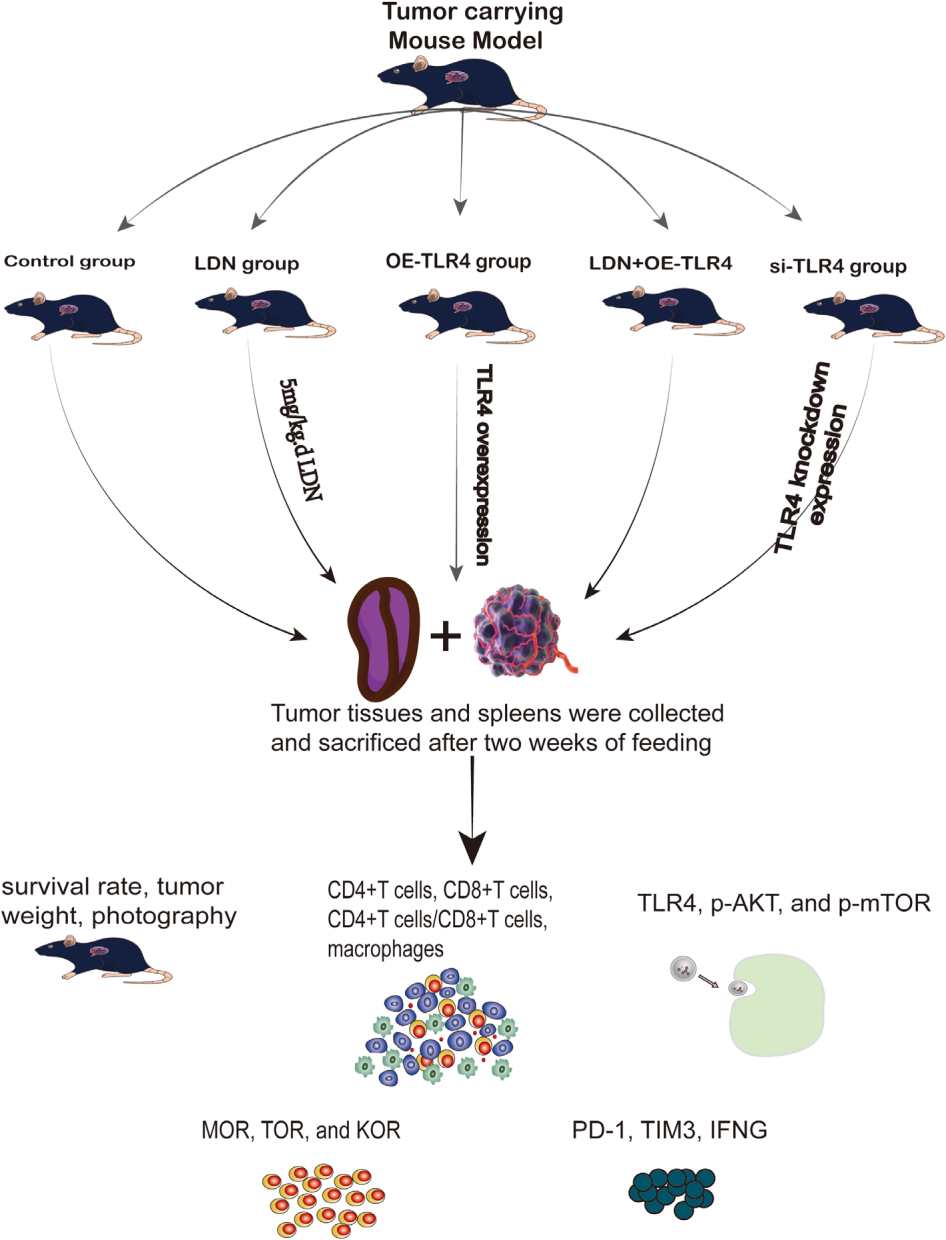
Flowchart of animal experiments.

(a) Construction of an MFC tumor-bearing mouse model: MFC cell suspension was diluted to a concentration of 2 × 10^7^ cells/mL and injected (0.1 mL) into the right axilla of C57BL/6 mice. The model was considered to be successful when a rice grain-sized tumor was visible under the subcutaneous tissue in the right axilla of the mice after 5–7 days of maintenance under specific pathogen-free (SPF) conditions.(b) The mice were randomly divided into five groups as follows: control (2 weeks of continuous intraperitoneal injection of phosphate buffered saline [PBS]), LDN (administered naloxone [5 mg/kg] once daily from the day of successful modeling until the day of euthanasia [2 weeks later]), OE-TLR4 (TLR4 overexpression), si-TLR4 (tail vein injection of 100 µL of virus [titer of 1 × 10^8^ PFU/mL]), and LDN + si-TLR4 groups. (c) Survival of mice and tumor growth were monitored. The mice were euthanized after 2 weeks, and the tumor tissue and spleen were collected. (d) Survival rate of mice, tumor weight, tumor imaging, growth curve, weight of mice, and hematoxylin/eosin (HE) staining of the tumor were performed. Flow cytometry was used to detect the number of CD4^+^T, CD8^+^T, CD4^+^T/CD8^+^T cells, M1 (F4/80^+^CD11b^+^), and M2 (F4/80^+^CD206^+^) macrophages in the spleen tissue. Sorting of CD8^+^T cells was performed. PCR was used to analyze TLR4, AKT, and mTOR expression levels in CD8^+^T cells. PCR was performed to analyze MOR, TOR, and KOR expression levels in CD8^+^T cells. The immune loci including PD-1, TIM3, and interferon gamma (IFN-γ) were also examined (**Figure 2**).

### 2.4 Cell culture

Human peripheral blood CD8^+^ T cells were cultured in complete culture medium for human CD8^+^ T cells supplemented with 10% fetal bovine serum and 1% penicillin-streptomycin. Cells were incubated at 37°C with 5% CO_2_ in a humidified incubator. Mouse gastric cancer cells (Wuhan Pricella Biotechnology Co. Ltd., Wuhan, China) were cultured in complete culture medium for human MFC cells supplemented with 10% fetal bovine serum and 1% penicillin-streptomycin at 37°C with 5% CO_2_ in a humidified incubator.

### 2.5 MFC tumor carrying mouse model

Twenty male C57BL/6 mice (6 weeks old) in logarithmic growth phase were used for construction of the MFC tumor-bearing mouse model. MFC cell concentration was adjusted to 2 × 10^7^ cells/mL in PBS. A total of 0.1 mL of MFC cell suspension was injected into the right axilla of mice under SPF conditions. The model was considered successful when rice grain-sized tumor (> 5 mm in diameter) was visible under the subcutaneous tissue in the right axilla of the mice after 5–7 days.

### 2.6 Transwell co-culture system of CD8^+^T and MFC cells

The transwell chambers were placed in a 24 well plate and the membrane was moistened with culture medium. In a cell culture incubator, the chamber was inverted with the bottom facing up, approximately 200 mL of MFC cell suspension was added, and incubated for 1–4 h at 37°C. The chamber was placed upright in the well in the culture plate, a layer of CD8^+^T cells was placed in the chamber, culture medium was added inside and outside the chamber, and the cells were incubated and allowed to adhere to the membrane.

### 2.7 Western blot (WB) assay

Proteins were extracted from the cells and immunoblotted, according to the manufacturer’s instructions. Horseradish peroxidase-conjugated secondary antibodies were used as appropriate. The primary antibodies used in this study are listed in **Supplementary Table S1**.

### 2.8 Cell transfection and lentiviral transduction

The siRNAs and plasmids used in this study were obtained from Shanghai Integrated Biotech Solutions Co. Ltd. The TLR4 overexpression plasmid and lipofectamine™ 2000 were purchased from Thermo Scientific. The plasmid for transfection was diluted with 250 μL Opti-Media to a final concentration of 50 μM, and mixed 3–5 times by gently pipetting. The transfection reagent and diluted plasmid solutions were mixed 3–5 times by gentle pipetting and incubated at room temperature for 20 min. The transfection mixture was then added to cells growing in a 6-well culture plate at 500 μL/well, and the plates were gently shaken for through mixing. PCR or WB assays were performed to analyze the data.

### 2.9 RNA extraction and quantitative realtime polymerase chain reaction (qRT-PCR)

Total RNA was extracted from cultured cells or tissues using TRIzol^®^ reagent. Total RNA was reverse transcribed into cDNA using the PrimeScript RT Reagent Kit according to the manufacturer’s instructions. Gene expression was analyzed using SYBR^®^ Green Master Mix, quantitated using the ΔΔCT method, and normalized to that of β-actin.

### 2.10 Flow cytometry

Flow cytometry and fluorescence-activated cell sorting were performed as described previously (20). Ischemic spleen tissues were dissociated into a single-cell suspension using 1 mg/mL collagenase and 0.1 mg/mL DNase I according to the manufacturer’s instructions. The cells were then stained with the indicated antibodies (diluted in 100 μL PBS) for 30 min at 4°C. The antibodies used in this study are listed in **Supplementary Table S1**. Cell acquisition was performed immediately after staining using the FACSDiva software on a flow cytometer (BD FACSCanto II). The FlowJo (version 10.6.2) software was used to analyze the data.

### 2.11 ELISA

The levels of IFN-γ and TNF-α were measured using the corresponding ELISA kits according to the manufacturer’s instructions. The optical density was measured at 450 nm using a microplate reader.

### 2.12 Statistical analyses

GraphPad Prism (version 10.1.0; Dotmatics) was used for the statistical analysis and graphical presentation. Results are presented as means ± standard error of mean (SD; N = sample size). GraphPad was used to assess the statistical significance between the two groups based on the two-tailed Student’s t-test. Multiple groups were compared using one-way ANOVA. Statistical significance was set at *p* < 0.05.

## 3 Results

### 3.1 TLR4 strongly correlated with immune cell function in patients with gastric cancer

**Figure 3:**
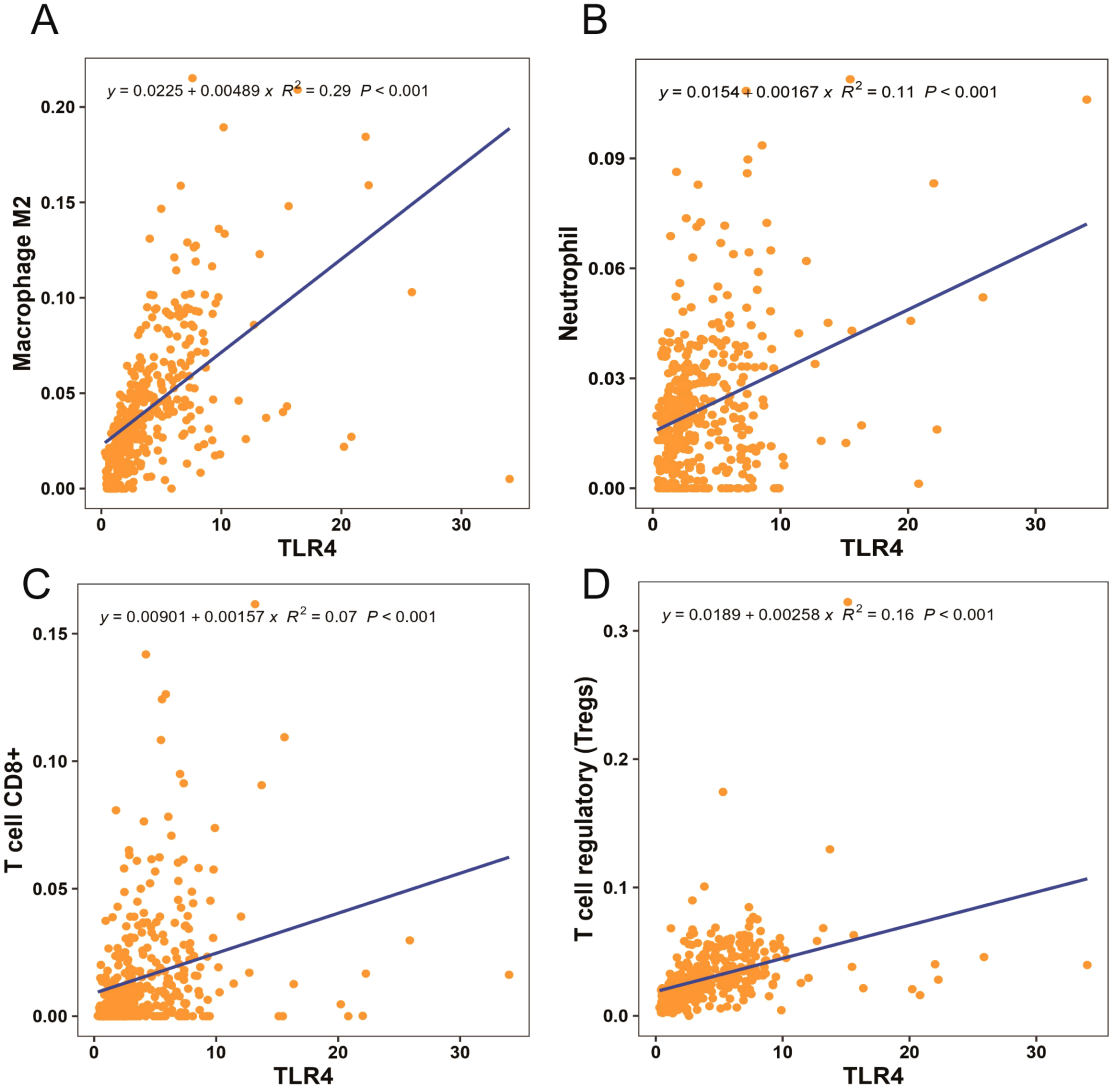
Macrophage M2, neutrophil, CD8^+^T, and regulatory T cell functions strongly correlated with toll-like receptor 4 (TLR4) signaling **(A–D)**.

We used TCGA database to examine the correlation between immune cell function and TLR4 in patients with gastric cancer. Analysis of macrophage, neutrophil, CD8^+^T, and regulatory T cell functions revealed a strong correlation with TLR4 signaling (**Figure 3**).

### 3.2 Naloxone increased the proliferation of CD8^+^ T cells and enhanced their ability to increases cytolytic protein expression

**Figure 4:**
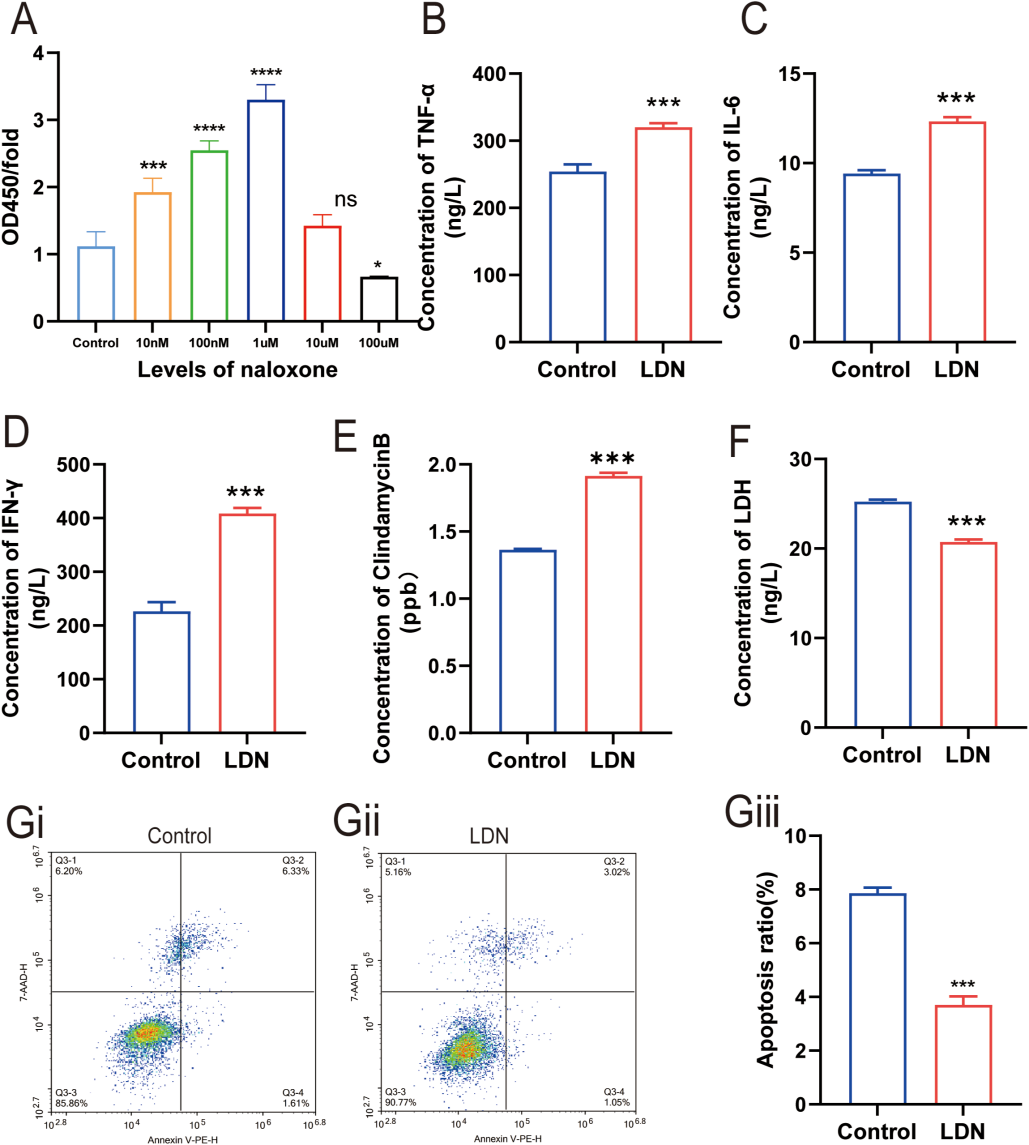
Effect of low-dose naloxone (LDN) on the cytolyticing and proliferative abilities of CD8^+^ T cells. **(A)** Cell Counting Kit-8 (CCK-8) assay was performed to analyze CD8^+^T cell proliferation following treatment with different concentrations of naloxone (10 nmol, 100 nmol, 1 µmol, 10 µmol, and 100 µmol) (N = 3)(One-way ANOVA). **(B-E)** ELISA was performed to determine the concentrations of TNFα, IL-6, IFN-γ, and clindamycin B in CD8^+^ T cells cultured in transwell co-culture systems (N = 3) (two-tailed Student’s *t*-test). **(F)** ELISA was performed to determine the concentrations of LDH in CD8^+^ T cells cultured in transwell co-culture systems (N = 3) (two-tailed Student’s *t*-test). **(Gi)** Apoptosis in the control group of CD8^+^ T cells cultured in transwell co-culture systems. **(Gii)** Apoptosis in the LDN group of CD8^+^ T cells cultured in transwell co-culture systems. **(Giii)** Quantitative evaluation of the number of CD8^+^ T cells (N = 3) (two-tailed Student’s *t*-test). LDN, low-dose naloxone (1 μmol). Each value represents the mean (N = 3) ± standard error of mean (SEM). *P < 0.05, ***P < 0.001 vs. control group. ns, no significance.

To examine the effect of different concentrations of naloxone on CD8^+^T cell proliferation, we used CCK8 assay to analyze CD8^+^T cell proliferation following treatment with different naloxone concentrations (10 nmol, 100 nmol, 1 μmol, 10 μmol, and 100 μmol). The results revealed that 1 μm naloxone strongly stimulated CD8^+^ T cells proliferation (**Figure 4A**). We treated CD8^+^ T cells in transwell co-culture systems with 1 μm naloxone and analyzed changes in the secretion of TNF-α, IL-6, IFN-γ, and GZMB. TNF-α, IL-6, IFN-γ, and clindamycin B expression was higher in the LDN group compared with that in the control group (**Figures 4B–E**). To validate the effect of LDN on CD8^+^T cell activity, we measured changes in the apoptotic rate and LDH levels in CD8^+^T cell in transwell co-culture systems. We found that LDN reduced LDH expression (**Figure 4F**) and decreased the rate of apoptosis in CD8^+^T cells (**Figures 4Gi–Giii**).

### 3.3 LDN reduced TIM-3 expression in CD8^+^T cells

**Figure 5:**
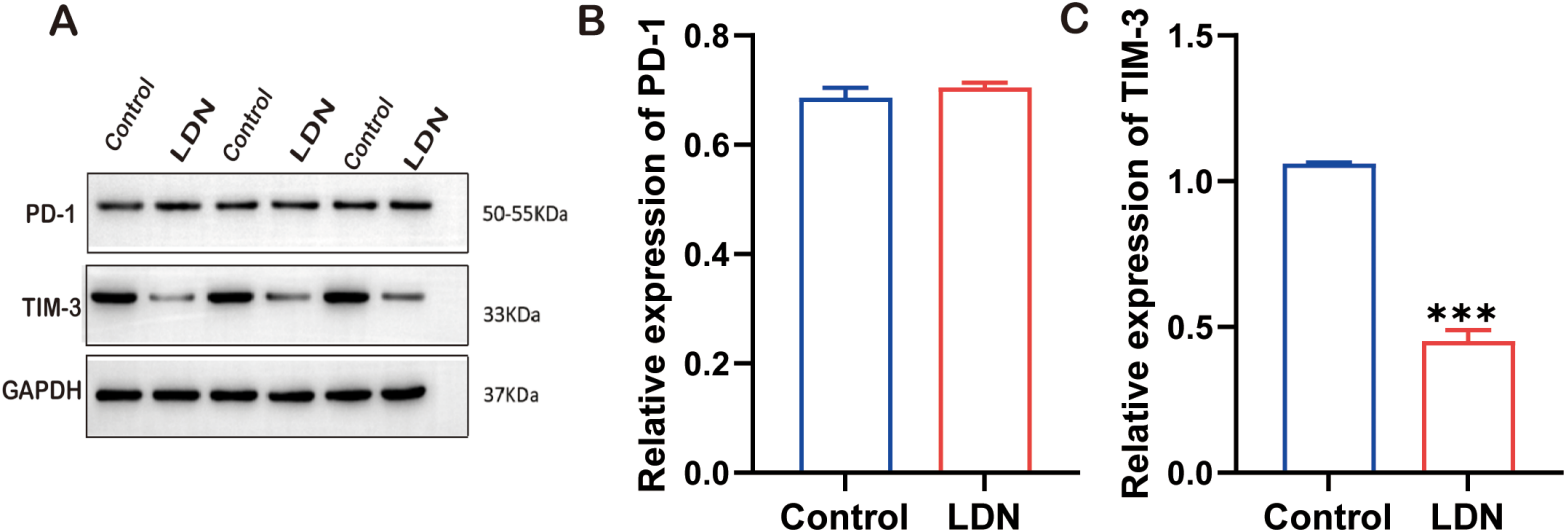
Programmed cell death -1 (PD-1), TIM-3, and GAPDH expression levels in CD8^+^ T cells cultured in transwell co-culture systems. **(A)** Western blotting of full-length TLR4, AKT, MOR, and GAPDH (N = 3). **(B)** Graph showing quantitation of PD-1 expression in CD8^+^T cells (two-tailed Student’s *t*-test). **(C)** Graph showing quantitation of TIM-3 expression in CD8^+^ T cells (two-tailed Student’s *t*-test). LDN, low-dose naloxone (1 μmol). Each value represents the mean (N = 3) ± standard error of mean (SEM). ***P < 0.001 vs. control group.

To further investigate the molecular mechanisms underlying the immune function changes in CD8^+^T cells, we examined the changes in PD-1 and TIM-3 expression following LDN treatment. The results demonstrated that LDN reduced TIM-3 expression in CD8^+^T cells (**Figures 5A–C**).

### 3.4 LDN reduced CD8^+^ T cells depletion and increased their cytotoxicity by regulating the TLR4/AKT/mTOR pathway

**Figure 6:**
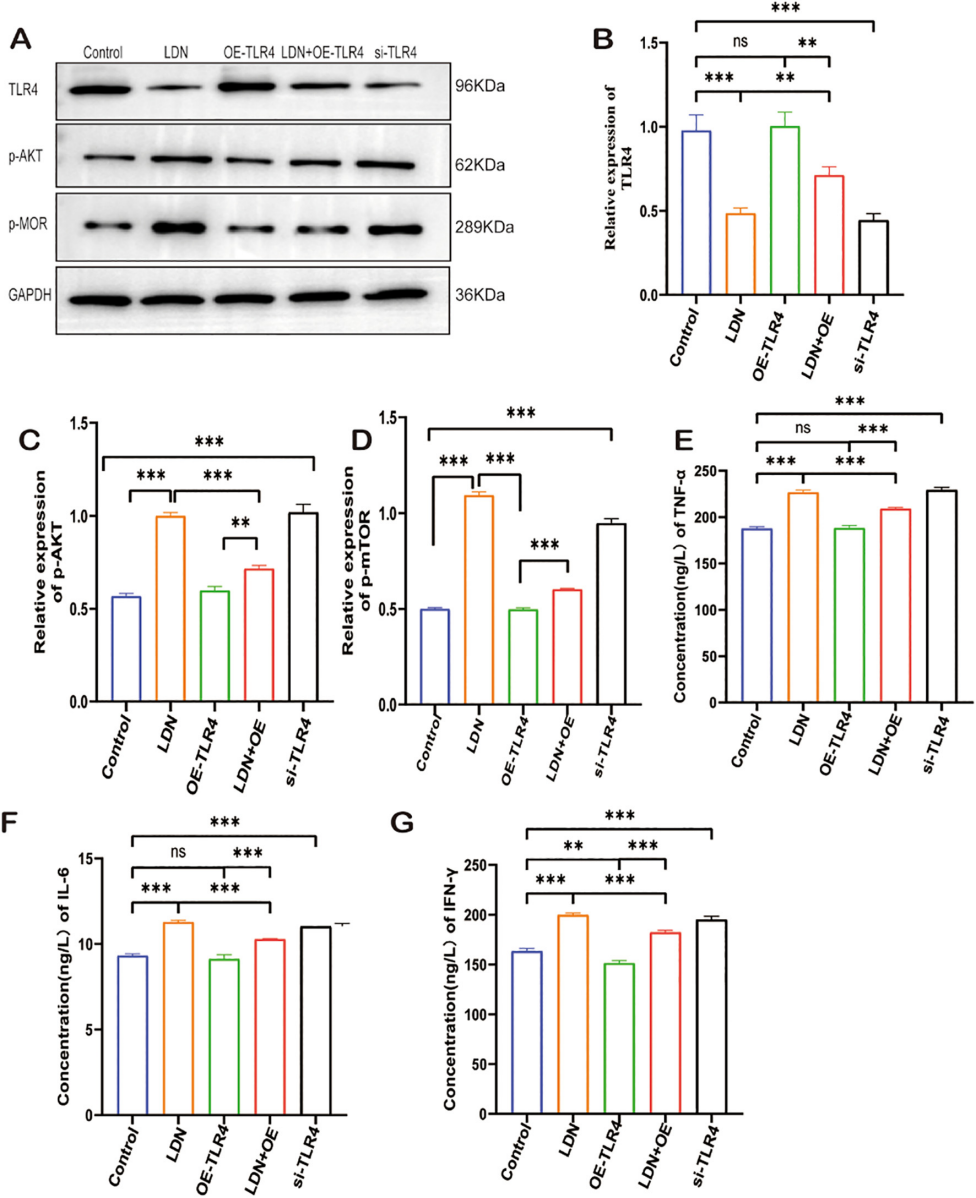
LDN reduces CD8^+^ T cell depletion in transwell co-culture systems and increases their cytotoxicity by regulating the TLR4/AKT/mTOR pathway. **(A)** Western blotting of full-length TLR4, AKT, MOR, and GAPDH (N = 3). **(B-D)** Graph showing quantitation of TLR-4/p-AKT/p-MOR expression in CD8^+^T cells, as determined by western blotting (N = 3)(One-way ANOVA). **(E-G)** Graph showing quantitation of TNF-α, IL-6, IFN-γ expression in CD8^+^T cells cultured in transwell co-culture systems as determined by ELISA (N = 3)(One-way ANOVA). LDN, low-dose naloxone (1 μmol). Each value represents the mean (N = 3) ± standard error of mean (SEM). **P < 0.01, ***P < 0.001. ns, no significance.

To investigate the specific molecular mechanisms underlying LDN-mediated effects on CD8^+^T cells, the transwell co-culture systems were randomly divided into five groups and treated as described in section 2.3.1. WB results revealed that TLR4 expression level was lower in the LDN group compared with that in the control group, whereas the OE-TLR4 group showed increased TLR4 expression. However, the results from the LDN + OE-TLR4 group suggested that LDN reduced the overexpression of TLR4 to a certain extent. In the si-TLR4 group, the results were consistent with those of the LDN treatment group, both of which showed reduced TLR4 expression in CD8^+^ T cells (**Figures 6A, B**). Compared to the control group, LDN treatment increased p-AKT and p-MOR expression levels. The results of the OE-TLR4 group were the opposite of those of the LDN group. However, when LDN was added to the OE-TLR4 group, the expression levels of p-AKT and p-MOR increased to a certain extent, whereas in si-TLR4 group, the expression levels of p-AKT and p-mTOR increased (**Figures 6C, D**). To further investigate the changes in the CD8^+^T cell-killing ability, we examined the changes in cytotoxic factors secreted by CD8^+^T cells. The results showed that LDN increased the secretion of TNF-α, IL-6, and IFN-γ by CD8^+^T cells compared with the OE-TLR4 group. The LDN + OE-TLR4 group also showed increased TNF-α, IL-6, and IFN-γ secretion. The results of the si-TLR4 group was consistent with that of the LDN group (**Figures 6E–G**).

### 3.5 LDN inhibited the expression of Lag3, Prf1, and Tim3 in CD8+ T cells

**Figure 7:**
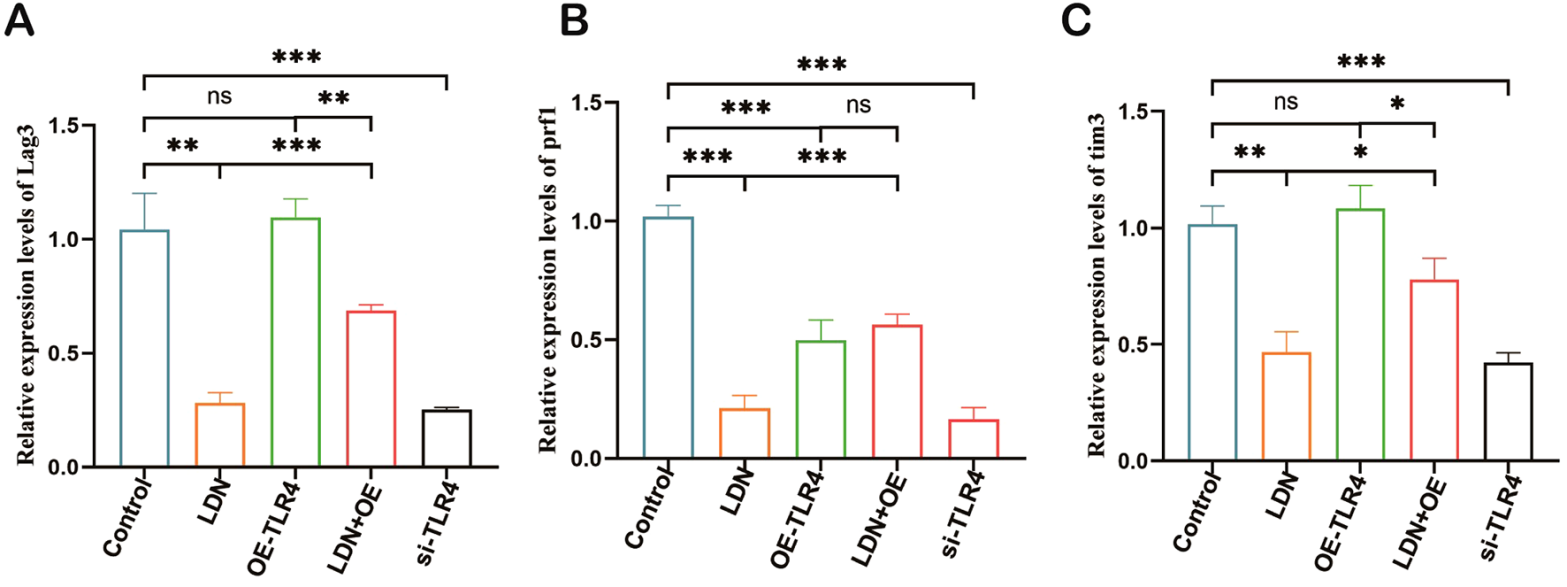
LDN inhibits the expression of Lag3, Prf1, and Tim3 in CD8^+^ T cells cultured in transwell co-culture systems. **(A-C)** Changes in Lag3, Prf1, and Tim3 expression levels in CD8^+^T cells cultured in transwell co-culture systems were determined using qRT-PCR. (N = 3)(One-way ANOVA), LDN, low-dose naloxone (1 μmol). Each value represents the mean ± standard error of mean (SEM) (N = 3). *P < 0.05, **P < 0.01, ***P < 0.001. ns, no significance.

A previous study indicated a strong correlation between immune checkpoints and CD8^+^T cell function (16). Therefore, we investigated the expression of Lag3, Prf1, and Tim3 in CD8^+^T cells. The results showed that LDN reduced the expression of Lag3, Prf1, and Tim3, whereas OE-TLR4 increased the expression of Lag3, Prf1, and Tim3 in CD8^+^T cells. In the LDN + OE-TLR4 group, LDN decreased the expression of Lag3, Prf1, and Tim3, whereas in the si-TLR4 group, TLR4 knockdown reduced the expression of Lag3, Prf1, and Tim3 (**Figures 7A–C**).

### 3.6 LDN reduced the tumor volume in MFC tumor-bearing mice and regulated the number of immune cells in the spleen

**Figure 8:**
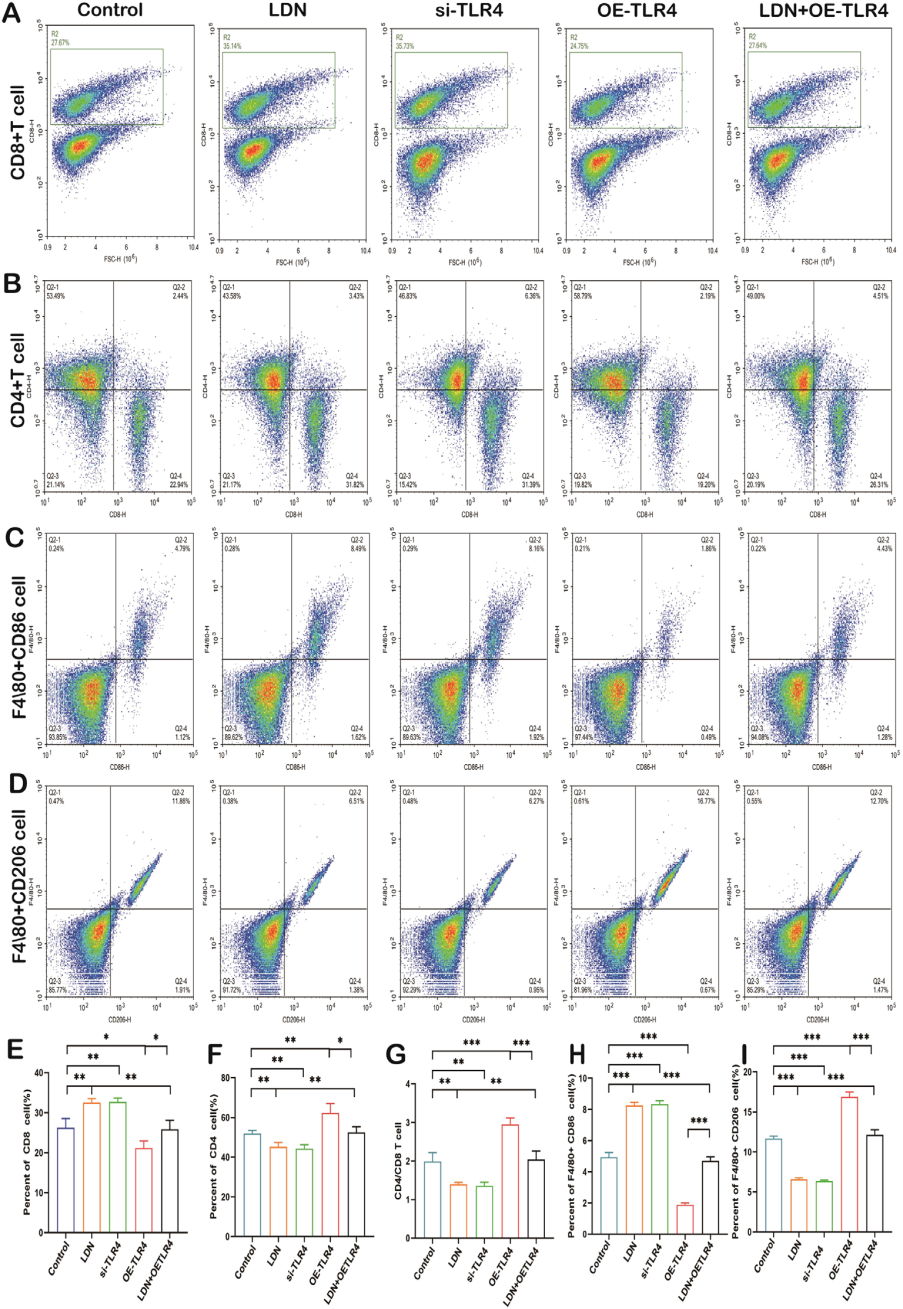
LDN increases the number of CD8^+^T cells and M1 macrophages, but decreases the number CD4^+^T cells, CD4/CD8^+^T, and M2 macrophages in the spleen. Flow cytometry was used to analyze the number of CD8^+^T **(A)**, CD4^+^T **(B)**, F4/80^+^CD86 **(C)**, and F4/80^+^CD206 **(D)** cells in the spleen (N = 4). Graph showing quantitation of CD8^+^ T cell **(E)**, CD4^+^T **(F)**, CD4/CD8^+^ T **(G)**, F4/80^+^CD86 **(H)**, and F4/80^+^CD206 **(I)** cell count in the spleen (N = 4). LDN was injected intraperitoneally at a dose of 5 mg/kg/day naloxone for two weeks. One-way ANOVA test with Tukey’s *post hoc* test, Each value represents the mean ± standard error of mean (SEM). *P < 0.05, **P < 0.01, ***P < 0.001.

Studies have demonstrated (21) that immune cells involved in innate and adaptive immunity play an important role in the microenvironment of tumors. Therefore, we performed *in vivo* experiments to observe changes in the number of various immune cells. The si-TLR4 group showed reduced tumor mass when compared with the control group, the OE-TLR4 group showed significantly increased tumor mass when compared with the control group, and LDN + OE-TLR4 group showed significantly reduced tumor mass when compared with the OE-TLR4 group (data are shown in **Supplementary Figure S1**). LDN treatment increased CD8^+^T cell numbers compared with that in the control group, and the results obtained from the si-TLR4 group were in agreement with those obtained from the LDN group. The number of CD8^+^T cells in the OE-TLR4 group was reduced compared with that in the control group. The LDN + OE-TLR4 group showed slightly increased number of CD8^+^T cells compared with that in the OE-TLR4 group (**Figures 8A, E**). The results obtained for M1 macrophages (F4/80^+^CD86 cells) were consistent with those obtained for CD8^+^ T cells (**Figures 8C, H**). Data on CD4^+^T cells numbers revealed that LDN slightly decreased the number of CD4^+^T cells. The si-TLR4 group showed a significant reduction in CD4^+^T cells compared to the control group. The number of CD4^+^T cells increased significantly in the OE-TLR4 group; however, compared to the OE-TLR4 group, there was a reduction in the number of CD4^+^T cells in the LDN + OE-TLR4 group (**Figures 8B, F**). A similar trend was observed in the number of CD4^+^/CD8^+^ T cells and M2 macrophages (F4/80^+^CD206 cells) (**Figures 8D, G, I**).

### 3.7 LDN increased the killing ability of CD8^+^ T cells

**Figure 9:**
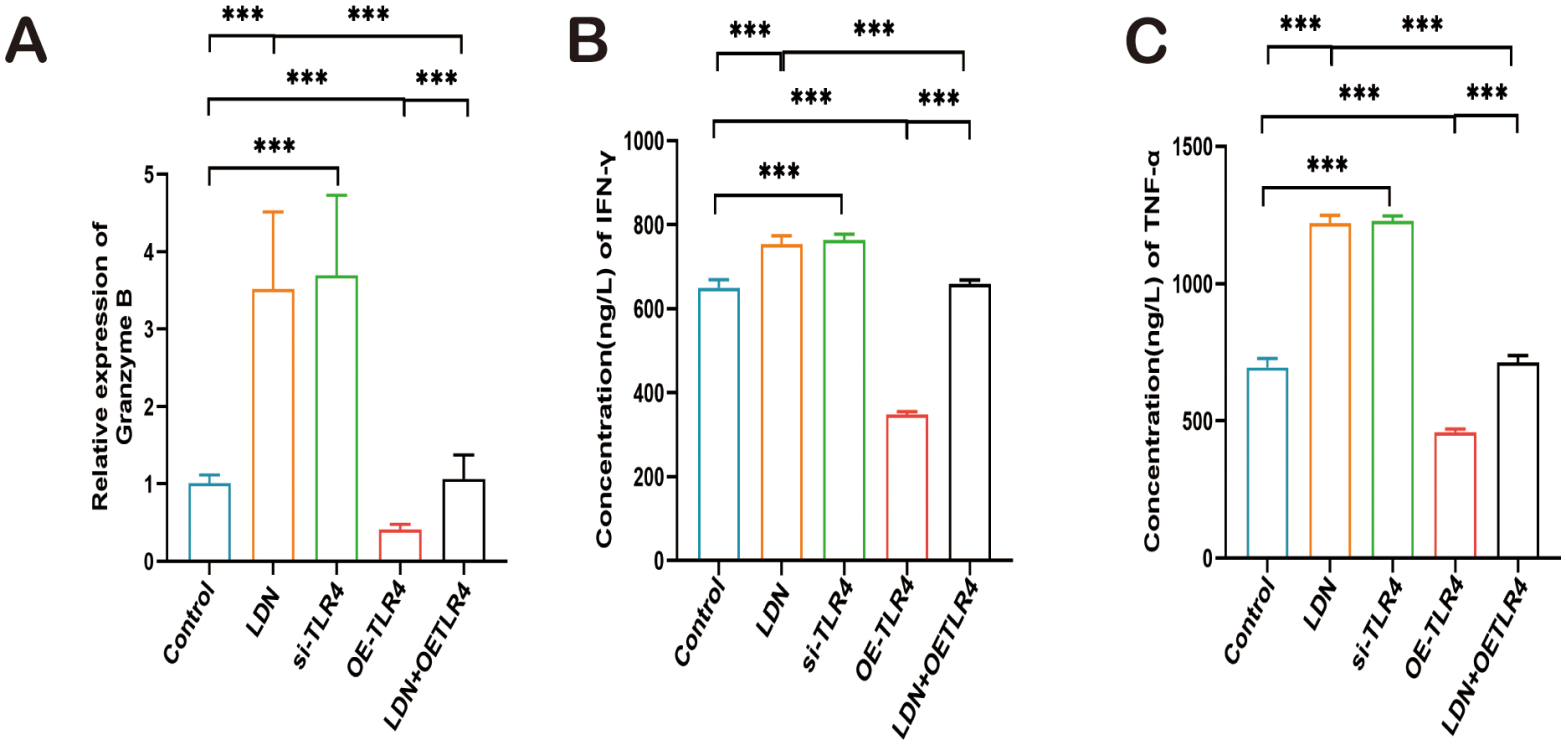
LDN increases granzyme B (GZMB), IFN-γ, and TNF-α secretion by CD8^+^T cells in the spleen. **(A)** ELISA was performed to analyze GZMB expression in CD8^+^T cells isolated from mouse spleen (N = 4). **(B, C)** qRT-PCR was performed to analyze IFN-γ and TNF-α expression in CD8^+^T cells isolated from mouse spleen (N = 4). LDN was injected intraperitoneally at a dose of 5 mg/kg/day naloxone for two weeks. One-way ANOVA test with Tukey’s *post hoc* test, Each value represents the mean (N = 4) ± standard error of mean (SEM). *P < 0.05, **P < 0.01, ***P < 0.001.

Next, we examined the changes in levels of granzyme B, IFN-γ, and TNF-α, which are important cytotoxic factors secreted by CD8^+^ T cells. A significant increase in granzyme B, IFN-γ, and TNF-α expression was observed in the LDN group. The results obtained from the si-TLR4 group and the LDN group were similar. The expression levels of granzyme B, IFN-γ, and TNF-α were significantly decreased in the OE-TLR4 group compared with that in the control group. In the LDN + OE-TLR4 group, LDN reversed the decline in granzyme B, IFN-γ, and TNF-α caused by OE-TLR4 (**Figures 9A–C**).

### 3.8 LDN decreased the expression of opioid receptors in CD8^+^ T cells

**Figure 10:**
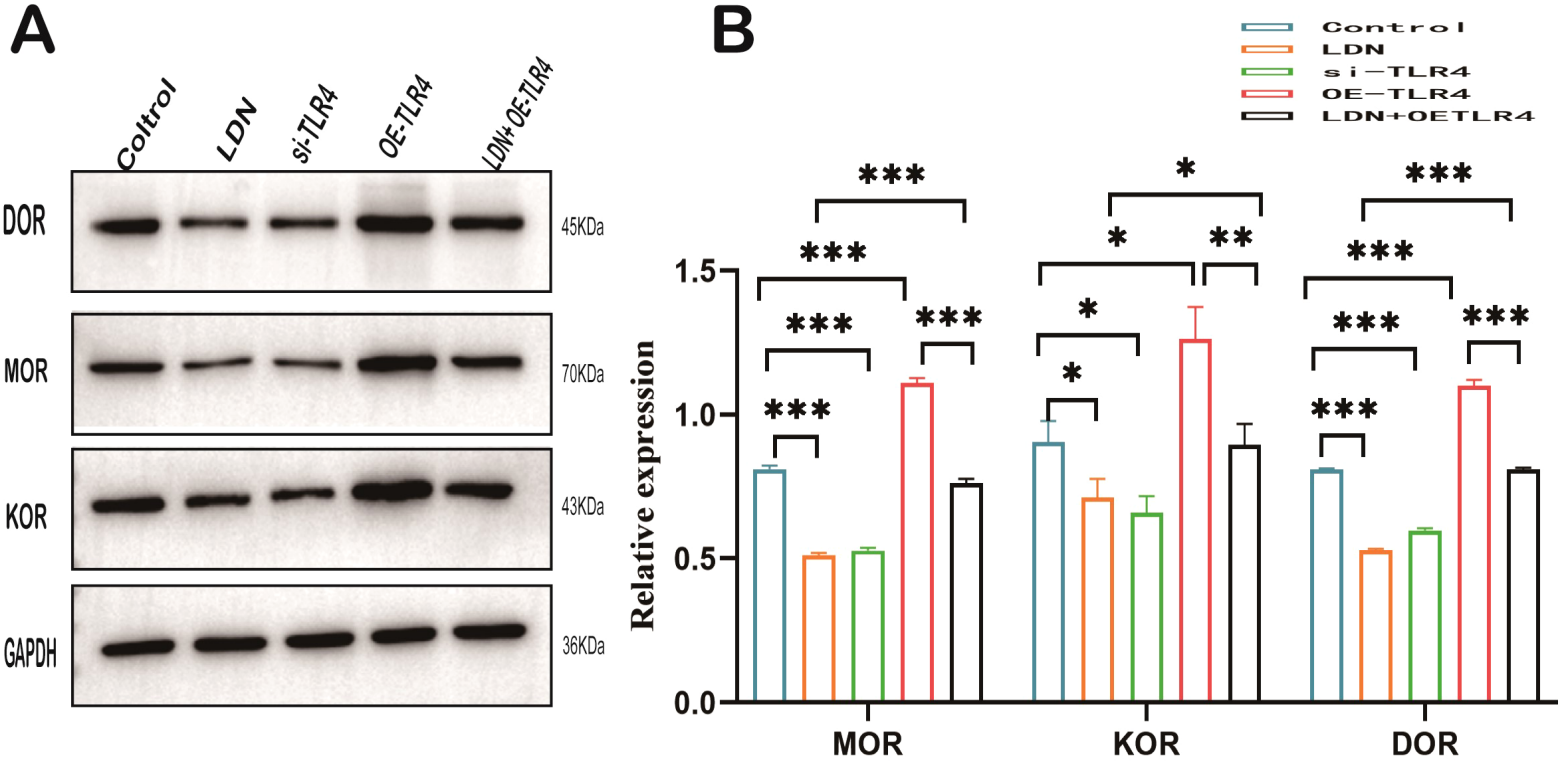
LDN reduces opioid receptor expression in CD8^+^ T cells in the spleen. **(A)** MOR, DOR, and KOR expression levels in CD8^+^ T cells were determined by western blotting (N=4). **(B)** Graph showing quantitation of MOR, DOR, and KOR expression in CD8^+^T cells determined by western blotting (N = 4). LDN was injected intraperitoneally at a dose of 5 mg/kg/day naloxone for two weeks. One-way ANOVA test with Tukey’s *post hoc* test, Each value represents the mean (N = 4) ± standard error of mean (SEM). *P < 0.05, **P < 0.01, ***P < 0.001.

Opioid receptors such as µ (MOR), δ (DOR) and κ (KOR) are closely related to immune cell function (22). Therefore, we examined the changes in the expression of these opioid receptors in CD8^+^T cells. The results revealed that the expression levels of µ, δ, and κ decreased in the LDN and si-TLR4 groups compared with those in the control group. µ, δ, and κ expression levels were significantly increased in the OE-TLR4 group, whereas, and results from the LDN + OE-TLR4 group demonstrated that LDN inhibited the increased expression of µ, δ, and κ caused by OE-TLR4 (**Figures 10A, B**).

### 3.9 LDN may alter the immune function of CD8+T cells by regulating the AKT/mTOR pathway

**Figure 11:**
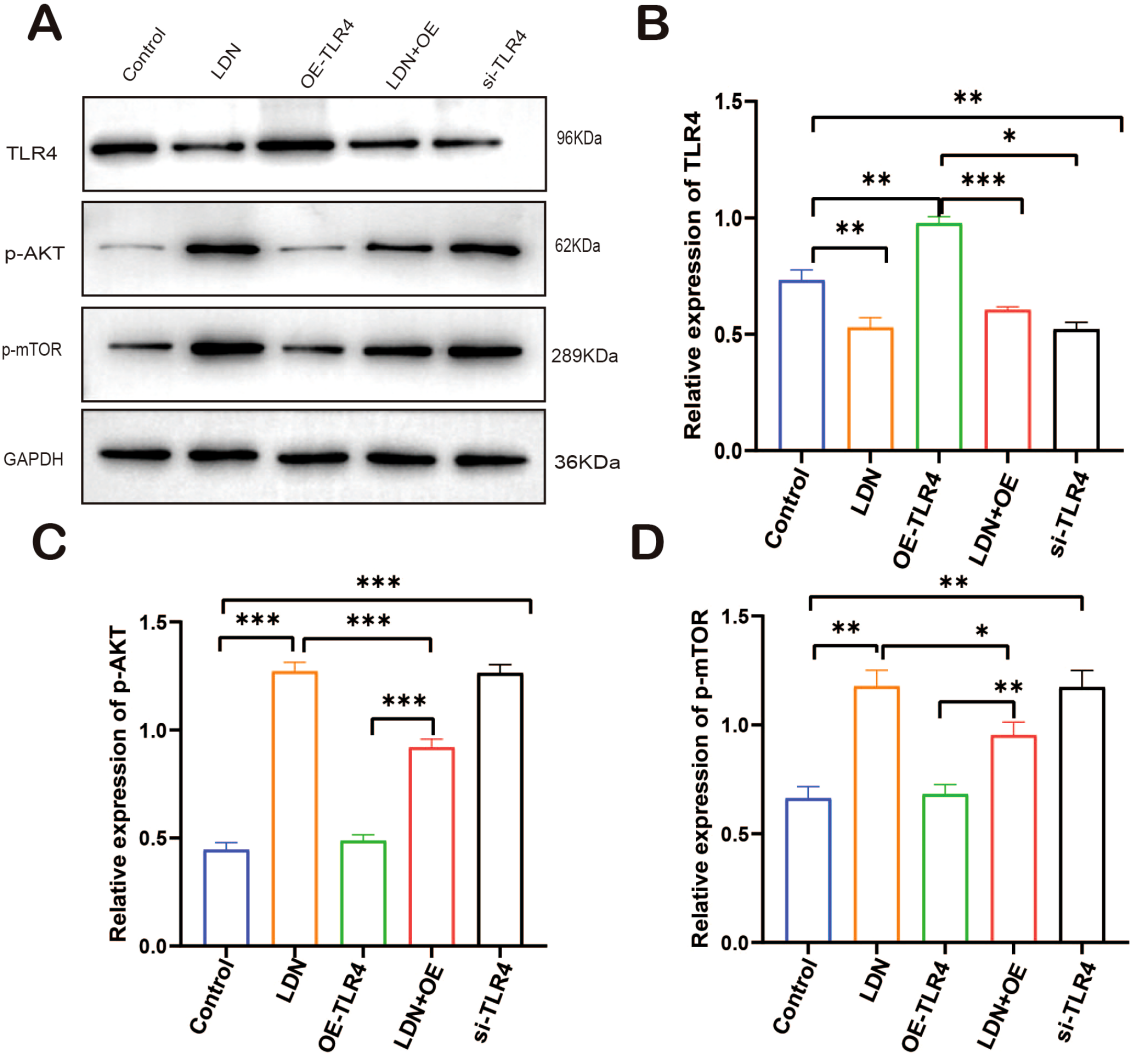
LDN increases AKT/mTOR expression in CD8^+^T cells in the spleen. **(A)** Western blot of full-length TLR4, AKT, mTOR, and GAPDH (N = 4 biological replicates). **(B)** Quantitative analysis of TLR4 expression in CD8^+^T cells. **(C)** Quantitative analysis of p-AKT expression in CD8^+^T cells. **(D)** Quantitative analysis of p-mTOR expression in CD8^+^T cells. LDN was injected intraperitoneally at a dose of 5 mg/kg/day naloxone for two weeks. One-way ANOVA test with Tukey’s *post hoc* test, Each value represents the mean (N = 4) ± standard error of mean (SEM). *P < 0.05, **P < 0.01, ***P < 0.001.

To further investigate the mechanisms underlying the immune function changes in CD8^+^T cells, we analyzed the changes in the expression of TLR4/AKT/mTOR pathway-related proteins. LDN increased AKT/mTOR expression in CD8^+^T cells, and the results obtained in the si-TLR4 group were similar to those obtained in the LDN group. AKT/mTOR expression was significantly downregulated in the OE-TLR4 group compared with that in the control group; the results from the LDN + OE-TLR4 group indicated that LDN inhibited the downregulation of AKT/mTOR expression caused by OE-TLR4 (**Figures 11A–D**).

### 3.10 LDN may improve the immune function of CD8^+^T cells by regulating the expression of immune checkpoint proteins

**Figure 12:**
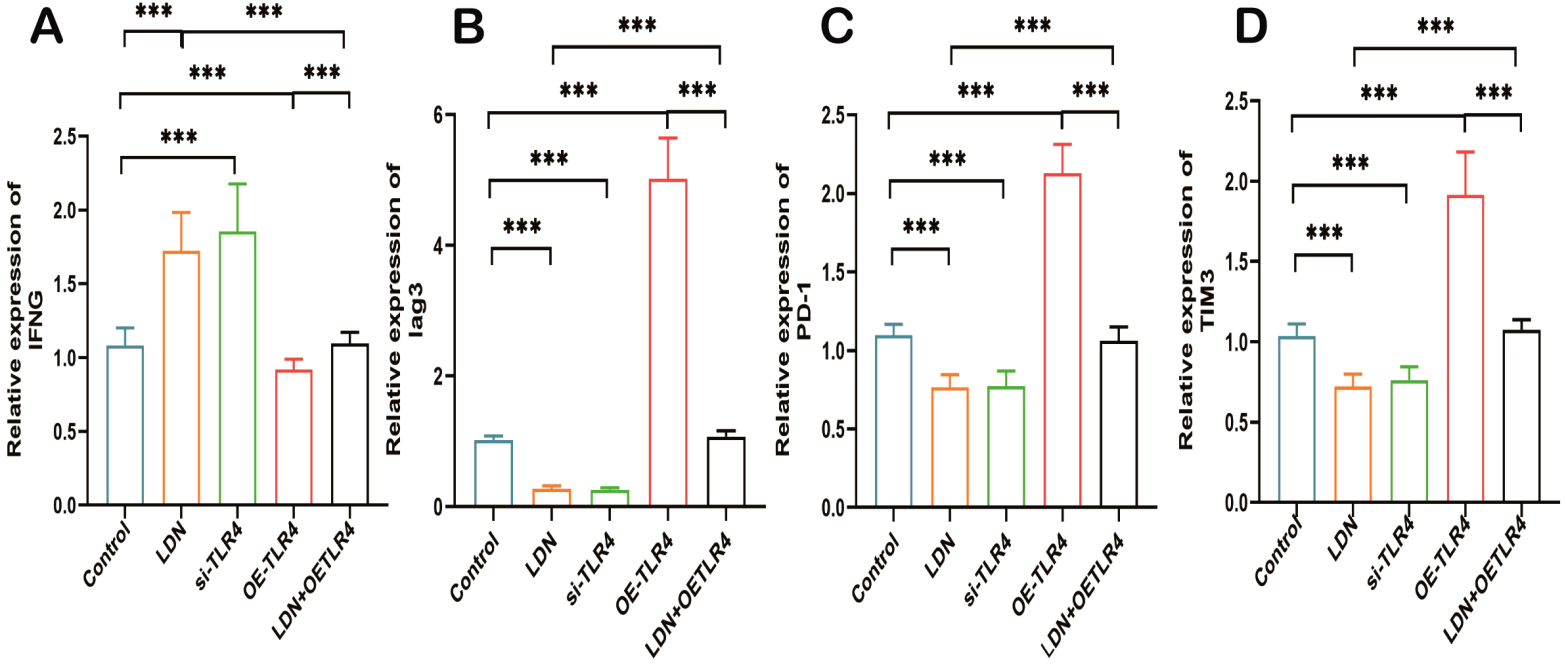
LDN increases IFN-γ expression and decreases Lag3, PD-1, and TIM3 expression in CD8^+^ T cells in the spleen. **(A)** Quantitative analysis of IFN-γ expression in CD8^+^T cells using qRT-PCR (N = 4). **(B)** Quantitative analysis of Lag3 expression in CD8^+^T cells using qRT-PCR (N = 4). **(C)** Quantitative analysis of PD-1 expression in CD8^+^T cells (N = 4) **(D)** Quantitative analysis of TIM3 expression in CD8^+^T cells (N = 4). LDN was injected intraperitoneally at a dose of 5 mg/kg/day naloxone for two weeks. One-way ANOVA test with Tukey’s *post hoc* test, Each value represents the mean (N=4) ± standard error of mean (SEM). *P < 0.05, ***P < 0.001, ****P < 0.0001.

LDN increased the expression of IFN-γ and decreased the expression of Lag3, PD-1, and TIM3 compared with that in the control group. The si-TLR4 group results were similar to that from the LDN group. The OE-TLR4 group showed reduced expression of IFN-γ and increased expression of Lag3, PD-1, and TIM3. In addition, the results from the LDN + OE-TLR4 group revealed that LDN reversed OE-TLR4-mediated decrease in IFN-γ and increase in Lag3, PD-1, and TIM3 expression (**Figures 12A–D**).

## 4 Discussion

Among malignant tumors, gastric cancer remains the most common and leading cause of cancer-related deaths worldwide, with some developing countries experiencing an increase in gastric cancer incidence and mortality in young patients. Surgery-based comprehensive treatment is the foundation of gastric cancer treatment and the only method that can completely eradicate gastric cancer lesions (23). Previous studies have suggested that LDN is a promising immune regulatory agent for the treatment of cancer and other diseases associated with the immune system (11). In addition, we previously demonstrated that perioperative administration of LDN reduces postoperative infection-related complications in patients with gastric cancer (17). In the present study, we examined the specific molecular biological mechanisms by which LDN improves immune function in patients with gastric cancer.

Numerous studies (24–26) have shown that the TLR4 pathway is involved in the occurrence and development of gastric cancer. It plays a crucial role in the tumor microenvironment in gastric cancer. Despite this, few studies have examined the relationship between changes in immune function and the TLR4 pathway in patients with perioperative gastric cancer. As a first step in ensuring that the experimental design of this study was feasible, we analyzed the correlation between the function of various immune cells and TLR4 in patients with gastric cancer from TCGA database. We found that macrophage M2, neutrophil, CD8^+^T, and regulatory T cell functions highly correlated with TLR4 signaling in the immune cells of patients with gastric cancer. Furthermore, previous studies (27) have demonstrated that CD8^+^T cells play a crucial role in the immune activity that kills tumor cells. We chose to focus on CD8^+^ T cells for further analyses.

Different concentrations of naloxone have been shown to have opposite effects on tumor cells. For example, LDN inhibits tumor growth, whereas high-dose naloxone accelerates tumor growth and somatic cell development (11). However, to date, no study has examined the optimal concentration of naloxone for improving CD8^+^T cells in *in vitro* experiments. In this study, for the first time, we demonstrate that 1 μm of naloxone strongly stimulates the proliferation of CD8^+^T cells. Intraperitoneal injection (28) of LDN (5 mg/kg) was reported to significantly improve the immune function in mice. Therefore, we chose an LDN dose of 5 mg/kg for the present study. Studies (14, 29) have shown a close functional relationship between opioid receptors and CD8^+^ T cells. The results of the present study demonstrated that LDN reduces the apoptotic rate of CD8^+^T cells and tumor volume in mice with gastric cancer. This is consistent with the results of previous studies (30)that LDN slows tumor growth in cancer patients, reduces long-term tumor recurrence rates, and improves CD8^+^T function. Upon binding to MHC-I antigens, CD8^+^T cell receptors trigger the differentiation and proliferation of cytotoxic T lymphocytes (CTLs). CTLs release cytotoxic substances such as TNF-α, IL-6, IFN-γ, and GZMB through their surface cytotoxic particles, directly killing tumor cells (31). In the present study, we have found that LDN increases the expression of TNF-α, IL-6, IFN-γ, and GZMB. Thus, LDN increases the tumor cell killing ability of CD8^+^T cells through some potential mechanism. Similar results have been reported in previous studies (32).

The immune checkpoint is regulated by programmed cell death protein 1 (PD-1) and its ligands. In recent years, immunotherapy has become a promising cancer treatment modality that stimulates the immune system to combat diseases. Immune checkpoint blockade constitutes one of the most effective immunotherapeutic strategies for the treatment of various cancers. By blocking the intrinsic down-regulators of immunity, such as cytotoxic T-lymphocyte antigen 4 (CTLA-4) and PD-1 or its ligands, programmed cell death ligand 1, the immune checkpoint blockade enhances antitumor immunity (33). Our study demonstrated the effects of LDN inhibitors on the expression of the programmed cell death receptors Lag3, Prf1, and Tim3 in CD8^+^T cells. These are expressed on the surface of T cells, whereas their ligands are expressed on the surface of tumor cells. The binding of programmed death receptors to their ligands results in the exhaustion of T cells, preventing the normal killing of tumor cells, which allows tumor cells to escape host immune surveillance. LDN inhibits the binding of these programmed death receptors and their ligands, enabling the host immune system to attack tumor cells more effectively. This may be one of the mechanisms through which LDN enhances the killing ability of CD8^+^ T cells.

T cells are involved in glucose metabolism via the AKT/mTOR signaling pathway. CD8^+^T cells are closely related to the TLR4/AKT/mTOR axis, and studies (34, 35) have shown that anti-TLR4 upregulates the expression of AKT/mTOR, further delaying the depletion of CD8^+^T cells. LDN increased the expression of p-AKT and p-mTOR, thereby delaying the loss of CD8^+^T cells, which may be another mechanism by which LDN enhances the killing ability of CD8^+^T cells.

Several studies (22) have shown that opioid receptors are widely expressed on T cells and involved in host immune and defense functions. According to a large body of literature (36–38), opioid drug abuse increases susceptibility to viral and bacterial infections. LDN represents a promising treatment option for immune-related diseases and cancers. The results of this study demonstrate that LDN reduces the expression of opioid receptors in CD8^+^T cells. This may be caused by the intermittent blockade of opioid receptors by LDN, which reduces tumor DNA synthesis and inhibits tumor growth (6). This may be one of the mechanisms by which LDN regulates CD8^+^T cell function.

## 5 Conclusion

This study is the first to demonstrate that LDN enhances the killing ability of CD8^+^T cells and reduces their exhaustion, thereby improving their ability to kill gastric cancer cells. Specifically, LDN may regulate the expression of immune checkpoints on CD8^+^ T cells, or increase the secretion of cytotoxic factors by CD8^+^T cells via the TLR4/AKT/mTOR pathway. LDN regulates the expression of opioid receptors on CD8^+^T cells, further affecting CD8^+^T cell exhaustion.

## Data Availability

The datasets presented in this study can be found in online repositories. The names of the repository/repositories and accession number(s) can be found in the article/**Supplementary Material**.

## References

[1] Smyth EC Nilsson M Grabsch HI van Grieken NC Lordick F. Gastric cancer. Lancet. (2020) 396:635–48. doi: 10.1016/S0140-6736(20)31288-5 32861308

[2] Karimi P Islami F Anandasabapathy S Freedman ND Kamangar F. Gastric cancer: descriptive epidemiology, risk factors, screening, and prevention. Cancer Epidemiol Biomarkers Prev. (2014) 23:700–13. doi: 10.1158/1055-9965.EPI-13-1057 PMC4019373 24618998

[3] Vallejo R Hord ED Barna SA Santiago-Palma J Ahmed S. Perioperative immunosuppression in cancer patients. J Environ Pathol Toxicol Oncol. (2003) 22:139–46. doi: 10.1615/JEnvPathToxOncol.v22.i2.70 14533877

[4] Zhang W Cong X Zhang L Sun M Li B Geng H. Effects of thoracic nerve block on perioperative lung injury, immune function, and recovery after thoracic surgery. Clin Transl Med. (2020) 10:e38. doi: 10.1002/ctm2.v10.3 32639645 PMC7418816

[5] Bhoir S Uhelski M Guerra-Londono JJ Cata JP. The role of opioid receptors in cancer. Adv Biol (Weinh). (2023) 7:e2300102. doi: 10.1002/adbi.202300102 37132160

[6] Hankins GR Harris RT. The opioid growth factor in growth regulation and immune responses in cancer. Adv Neurobiol. (2024) 35:45–85. doi: 10.1007/978-3-031-45493-6_4 38874718

[7] Mélik Parsadaniantz S Rivat C Rostène W Réaux-Le-Goazigo A. Opioid and chemokine receptor crosstalk: a promising target for pain therapy? Nat Rev Neurosci. (2015) 16:69–78. doi: 10.1038/nrn3858 25588373

[8] Li Y Sun L Zhou Q Lee AJ Wang L Zhang R. Effects of opioid drugs on immune function in cancer patients. BioMed Pharmacother. (2024) 175:116665. doi: 10.1016/j.biopha.2024.116665 38701564

[9] Sun W Zhuang S Cheng M Qiu Z. Mu opioid receptor mRNA overexpression predicts poor prognosis among 18 common solid cancers: A pan-cancer analysis. Front Oncol. (2023) 13:1134744. doi: 10.3389/fonc.2023.1134744 37064155 PMC10098160

[10] Giakomidi D Bird MF Lambert DG. Opioids and cancer survival: are we looking in the wrong place? BJA Open. (2022) 2:100010. doi: 10.1016/j.bjao.2022.100010 37588274 PMC10430855

[11] Li Z You Y Griffin N Feng J Shan F. Low-dose naltrexone (LDN): A promising treatment in immune-related diseases and cancer therapy. Int Immunopharmacol. (2018) 61:178–84. doi: 10.1016/j.intimp.2018.05.020 29885638

[12] Couto RD Fernandes BJD. Low doses naltrexone: the potential benefit effects for its use in patients with cancer. Curr Drug Res Rev. (2021) 13:86–9. doi: 10.2174/2589977513666210127094222 33504322

[13] Loizzo A Loizzo S Lopez L d'Amore A Renzi P Spampinato S. Naloxone prevents cell-mediated immune alterations in adult mice following repeated mild stress in the neonatal period. Br J Pharmacol. (2002) 135:1219–26. doi: 10.1038/sj.bjp.0704577 PMC1573241 11877330

[14] Skiba D Jaskuła K Nawrocka A Poznański P Łazarczyk M Szymański Ł. The role of opioid receptor antagonists in regulation of blood pressure and T-cell activation in mice selected for high analgesia induced by swim stress. Int J Mol Sci. (2024) 25 (5):2618. doi: 10.3390/ijms25052618 38473865 PMC10932203

[15] McLaughlin PJ Odom LB Arnett PA Orehek S Thomas GA Zagon IS. Low-dose naltrexone reduced anxiety in persons with multiple sclerosis during the COVID-19 pandemic. Int Immunopharmacol. (2022) 113:109438. doi: 10.1016/j.intimp.2022.109438 36379151 PMC9643313

[16] Gondoh E Hamada Y Mori T Iwazawa Y Shinohara A Narita M. Possible mechanism for improving the endogenous immune system through the blockade of peripheral μ-opioid receptors by treatment with naldemedine. Br J Cancer. (2022) 127:1565–74. doi: 10.1038/s41416-022-01928-x PMC9553910 35945243

[17] Min X Ma Y Leng Y Li X Zhang J Xu S. Effects of perioperative low-dose naloxone on the immune system in patients undergoing laparoscopic-assisted total gastrectomy: a randomized controlled trial. BMC Anesthesiol. (2024) 24:172. doi: 10.1186/s12871-024-02524-7 38720250 PMC11077871

[18] Yi Z Guo S Hu X Wang X Zhang X Griffin N. Functional modulation on macrophage by low dose naltrexone (LDN). Int Immunopharmacol. (2016) 39:397–402. doi: 10.1016/j.intimp.2016.08.015 27561742

[19] McIlvried LA Martel Matos AA Yuan MM Atherton MA Obuekwe F Nilsen ML. Morphine treatment restricts response to immunotherapy in oral squamous cell carcinoma. J Immunotherapy Cancer. (2004) 12(11):e009962. doi: 10.1136/jitc-2024-009962 PMC11574397 39551606

[20] An X Chen L. Flow cytometry (FCM) analysis and fluorescence-activated cell sorting (FACS) of erythroid cells. Methods Mol Biol. (2018) 1698:153–74. doi: 10.1007/978-1-4939-7428-3_9 29076089

[21] Gajewski TF Schreiber H Fu YX. Innate and adaptive immune cells in the tumor microenvironment. Nat Immunol. (2013) 14:1014–22. doi: 10.1038/ni.2703 PMC4118725 24048123

[22] Malik JA Agrewala JN. Morphine's role in macrophage polarization: exploring M1 and M2 dynamics and disease susceptibility. J Neuroimmunol. (2025) 400:578534. doi: 10.1016/j.jneuroim.2025.578534 39883986

[23] Yang WJ Zhao HP Yu Y Wang JH Guo L Liu JY. Updates on global epidemiology, risk and prognostic factors of gastric cancer. World J Gastroenterol. (2023) 29:2452–68. doi: 10.3748/wjg.v29.i16.2452 PMC10167900 37179585

[24] Zargari S Bahari A Goodarzi MT Mahmoodi M Valadan R. TLR2 and TLR4 signaling pathways and gastric cancer: insights from transcriptomics and sample validation. Iran BioMed J. (2022) 26:36–43. doi: 10.52547/ibj.26.1.36 34773930 PMC8784901

[25] Li Z Gao H Liu Y Wu H Li W Xing Y. Genetic variants in the regulation region of TLR4 reduce the gastric cancer susceptibility. Gene. (2021) 767:145181. doi: 10.1016/j.gene.2020.145181 33007372

[26] He B Xu T Pan B Pan Y Wang X Dong J. Polymorphisms of TGFBR1, TLR4 are associated with prognosis of gastric cancer in a Chinese population. Cancer Cell Int. (2018) 18:191. doi: 10.1186/s12935-018-0682-0 30479570 PMC6245525

[27] Giles JR Globig AM Kaech SM Wherry EJ. CD8(+) T cells in the cancer-immunity cycle. Immunity. (2023) 56:2231–53. doi: 10.1016/j.immuni.2023.09.005 PMC11237652 37820583

[28] Sacerdote P Gaspani L Panerai AE. The opioid antagonist naloxone induces a shift from type 2 to type 1 cytokine pattern in normal and skin-grafted mice. Ann N Y Acad Sci. (2000) 917:755–63. doi: 10.1111/j.1749-6632.2000.tb05440.x 11268404

[29] Baddack-Werncke U Busch-Dienstfertig M González-Rodríguez S Maddila SC Grobe J Lipp M. Cytotoxic T cells modulate inflammation and endogenous opioid analgesia in chronic arthritis. J Neuroinflamm. (2017) 14:30. doi: 10.1186/s12974-017-0804-y PMC5294766 28166793

[30] Amaram-Davila J Vega MF Kim MJ Dalal S Dev R Tanco K. Perceptions toward naloxone among patients with cancer receiving opioids. J Pain Symptom Manage. (2024) 68:e500–e7. doi: 10.1016/j.jpainsymman.2024.08.034 39218123

[31] St Paul M Ohashi PS. The roles of CD8(+) T cell subsets in antitumor immunity. Trends Cell Biol. (2020) 30:695–704. doi: 10.1016/j.tcb.2020.06.003 32624246

[32] Molla Hassan AT Hassan ZM Moazzeni SM Mostafaie A Shahabi S Ebtekar M. Naloxone can improve the anti-tumor immunity by reducing the CD4+CD25+Foxp3+ regulatory T cells in BALB/c mice. Int Immunopharmacol. (2009) 9:1381–6. doi: 10.1016/j.intimp.2009.08.008 19706340

[33] Postow MA Sidlow R Hellmann MD. Immune-related adverse events associated with immune checkpoint blockade. N Engl J Med. (2018) 378:158–68. doi: 10.1056/NEJMra1703481 29320654

[34] Zhou X Fang D Liu H Ou X Zhang C Zhao Z. PMN-MDSCs accumulation induced by CXCL1 promotes CD8(+) T cells exhaustion in gastric cancer. Cancer Lett. (2022) 532:215598. doi: 10.1016/j.canlet.2022.215598 35176418

[35] Wang B Li X Li M Geng Y Wang N Jin Y. Dopamine D3 receptor signaling alleviates mouse rheumatoid arthritis by promoting Toll-like receptor 4 degradation in mast cells. Cell Death Dis. (2022) 13:240. doi: 10.1038/s41419-022-04695-y 35292659 PMC8924203

[36] Rogers TJ Roy S. Editorial: the role of opioid receptors in immune system function. Front Immunol. (2021) 12:832292. doi: 10.3389/fimmu.2021.832292 35082800 PMC8784803

[37] Zhang P Yang M Chen C Liu L Wei X Zeng S. Toll-like receptor 4 (TLR4)/opioid receptor pathway crosstalk and impact on opioid analgesia, immune function, and gastrointestinal motility. Front Immunol. (2020) 11:1455. doi: 10.3389/fimmu.2020.01455 32733481 PMC7360813

[38] Rogers TJ. Kappa opioid receptor expression and function in cells of the immune system. Handb Exp Pharmacol. (2022) 271:419–33. doi: 10.1007/164_2021_441 33580386

