# Supplementary material for: Exploring the possible mechanism of low-dose naloxone exposure improving the immune microenvironment of gastric cancer tumors
Source: Front Immunol. 2025 Mar 26;16:1524930. doi: 10.3389/fimmu.2025.1524930 (PMC11979148; doi:10.3389/fimmu.2025.1524930)
Supplement: Supplementary file 1 [file Table1.docx]

Supplementary Material

# Supplementary Table

Supplementary Table 1 The primer sequences.

| Gene | Forward Primer | Reverse Primer |
| --- | --- | --- |
| GAPDH | GGTGAAGGTCGGTGTGAACG | CTCGCTCCTGGAAGATGGTG |
| AKT | GCCGCCTGATCAAGTTCTCC | TTCAGATGATCCATGCGGGG |
| mTOR | CACAAGGAGATCCGCATGGA | GCGGATATCAGGGTCAGGAT |
| TLR4 | TCCCTGCATAGAGGTAGTTCC | TCAAGGGGTTGAAGCTCAGA |
| GZMB | CTGCTCACTGTGAAGGAAGT | GGGATGACTTGCTGGGTCTT |
| IFNG | AGACAATCAGGCCATCAGCAA | GTGGGTTGTTGACCTCAAACT |
| lag3 | GGCGGTCATCACAGTGACTC | TTTCCAGATGCCGGGGTTAC |
| PD-1 | TGCCCTAGTGGGTATCCCTG | AAGGCTCCTCCTTCAGAGTG |
| TIM3 | AGACATCAAAGCAGCCAAGG | TCCGTGGTTAGGGTTCTTGG |
| prf1 | GAGCCTCGGTGAAGAGAGG | GGCACTTGGGCTCTGGAAT |

# Supplementary Figures


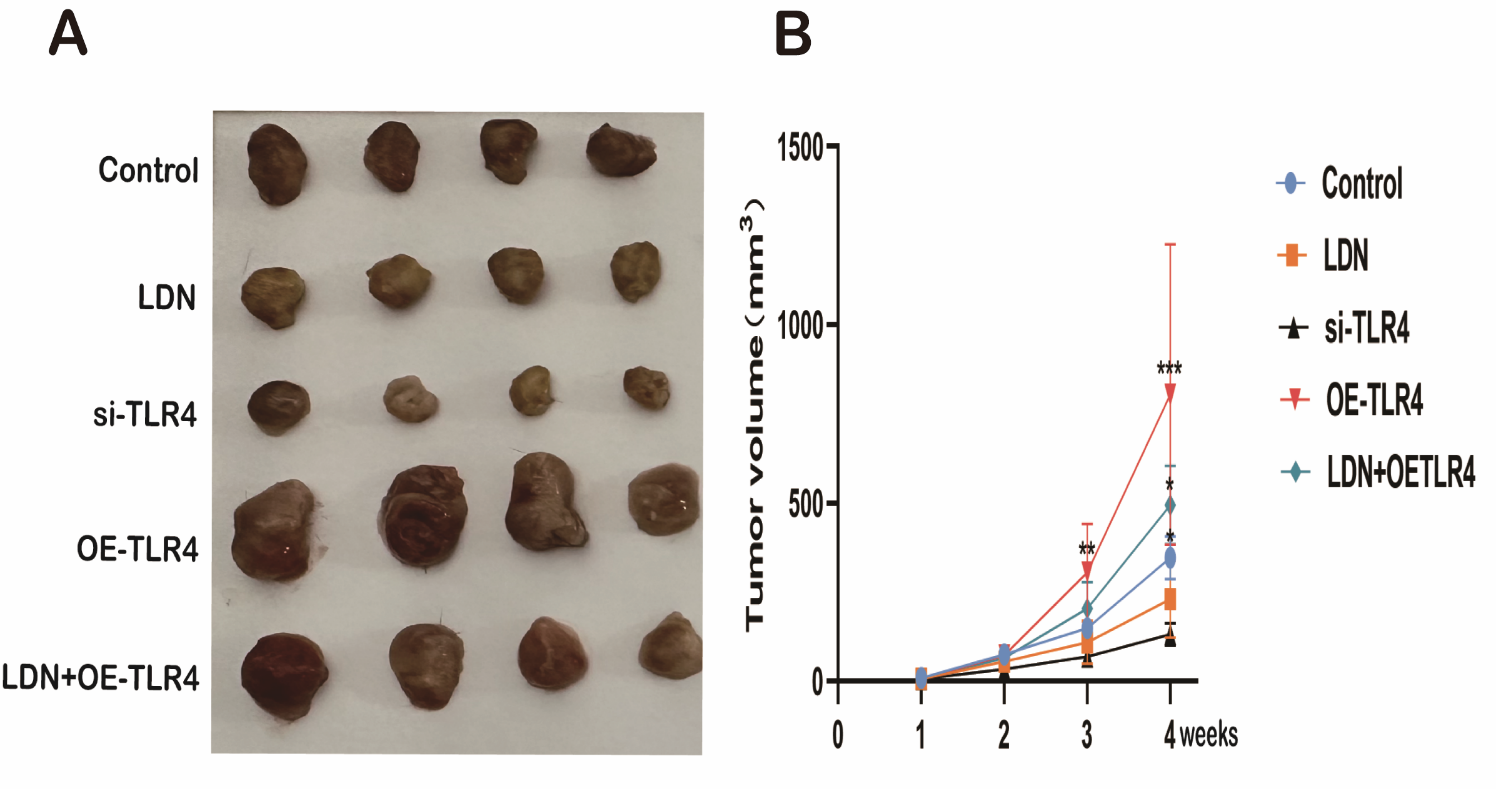


Supplementary Figure 1 A. Upon euthanizing the mice in the fourth week, the tumor was collected and photographed. B. Change in tumor volume from the first to the fourth week. **P* < 0.05, ***P* < 0.01,****P* < 0.001vs. the Control group.

**
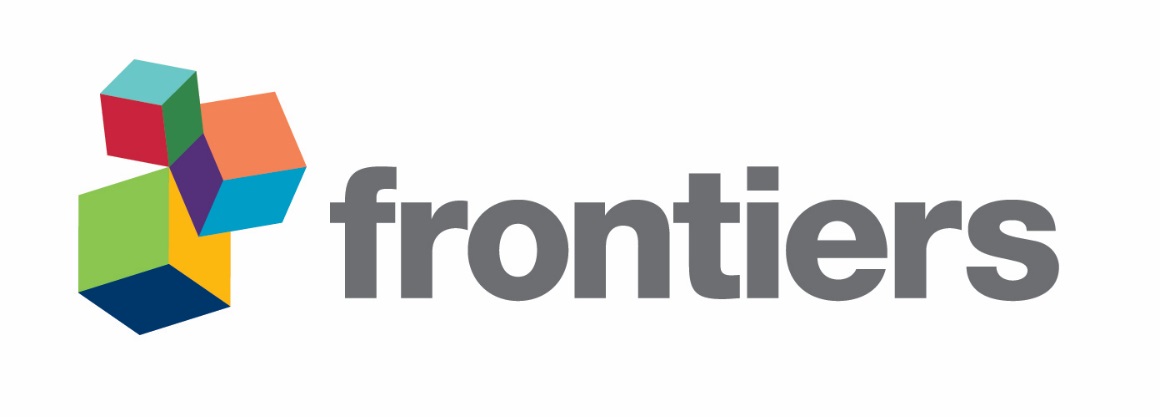
**
